# Multifocal Skeletal Tuberculosis Mimicking Metastatic Malignancy: A Case Report

**DOI:** 10.7759/cureus.111029

**Published:** 2026-06-17

**Authors:** Poleth G Medrano-Madrid, Edgar A Espinoza-Beltrán, Jose M Espinoza-Beltrán, Erika M Espinoza-Beltrán

**Affiliations:** 1 Internal Medicine, Servicios de Salud del Instituto Mexicano del Seguro Social para el Bienestar, Hospital General Culiacán, Culiacan, MEX; 2 Internal Medicine, Servicios de Salud del Instituto Mexicano del Seguro Social para el Bienestar, Hospital General Culiacán, Culiacán, MEX; 3 Radiology, Centro de Investigación y Docencia en Ciencias de la Salud, Hospital Civil de Culiacán, Culiacán, MEX; 4 Infectious Disease, Universidad Autónoma de Sinaloa, Culiacán, MEX

**Keywords:** extrapulmonary tuberculosis, metastatic malignancy, multifocal skeletal tuberculosis, osteolytic bone lesions, paraplegia, skeletal tuberculosis, spinal cord compression

## Abstract

Skeletal tuberculosis is an uncommon form of extrapulmonary tuberculosis that may involve multiple osseous sites and occasionally mimic malignant disease, creating a significant diagnostic challenge. We report the case of a 70-year-old male who presented with an eight-month history of progressive thoracolumbar pain, constitutional symptoms, and subsequent development of paraplegia and urinary retention. Computed tomography revealed multifocal osteolytic lesions involving the sternum, thoracic and lumbar vertebrae, and iliac bones, initially raising concern for metastatic malignancy. Magnetic resonance imaging demonstrated destructive thoracic spondylodiscitis with additional noncontiguous vertebral involvement and associated epidural and paravertebral extension resulting in spinal canal compromise. Definitive diagnosis was established through computed tomography-guided biopsy followed by conventional polymerase chain reaction, which detected *Mycobacterium tuberculosis *complex. Antituberculous therapy was initiated, and the patient was subsequently evaluated by the Neurosurgery service, where he was deemed a candidate for surgical decompression and is currently undergoing preoperative assessment. This case highlights the importance of considering multifocal skeletal tuberculosis in the differential diagnosis of multifocal osteolytic lesions suggestive of malignancy, particularly in endemic regions.

## Introduction

Tuberculosis (TB) remains a major global health problem, and extrapulmonary involvement continues to contribute substantially to disease burden, particularly in endemic regions [1]. Skeletal tuberculosis is an uncommon form of extrapulmonary TB that may involve virtually any bone or joint. Bone and joint tuberculosis accounts for approximately 11.3% of all tuberculosis cases, with its incidence among extrapulmonary tuberculosis cases ranging from 10% to 21.1% [2]. Among skeletal sites, the spine is the most frequently affected location and is associated with substantial morbidity because delayed diagnosis may result in spinal instability, deformity, and neurological deficits [2,3].

Multifocal skeletal tuberculosis is a rare manifestation characterized by involvement of multiple osseous sites, often presenting as multifocal osteolytic lesions. Because of its radiological appearance and widespread skeletal distribution, multifocal skeletal tuberculosis may closely resemble metastatic malignancy, creating a significant diagnostic challenge and frequently delaying definitive diagnosis [4]. Distinguishing multifocal skeletal tuberculosis from metastatic malignancy is clinically important because delayed recognition may result in disease progression, neurological compromise, and postponement of appropriate therapy [3,4].

We report the case of an elderly patient with multifocal skeletal tuberculosis involving the thoracolumbar spine, sternum, and iliac bones that initially mimicked metastatic malignancy and subsequently progressed to paraplegia.

## Case presentation

A 70-year-old male agricultural worker with a remote history of gastrojejunostomy for peptic ulcer disease presented with an eight-month history of progressive thoracolumbar pain. He had no known history of tuberculosis, malignancy, or chronic medical conditions.

The pain was initially mechanical in nature and progressively worsened despite treatment with multiple analgesic regimens. During the course of the illness, he developed constitutional symptoms including intermittent fever, nocturnal diaphoresis, and an unintentional weight loss of approximately 10 kg. Several febrile episodes were initially attributed to recurrent urinary tract infections.

Prior to neurological deterioration, the patient required a cane for ambulation and assistance with activities of daily living due to progressive pain and functional limitation. One week before admission, the patient experienced abrupt worsening of pain associated with progressive gait impairment, culminating in complete loss of motor function in both lower extremities. He also developed urinary retention requiring bladder catheterization. Neurological examination revealed paraplegia with bilateral lower-extremity strength graded as 0/5 according to the Medical Research Council (MRC) scale [5], absent patellar and Achilles reflexes, bilateral Babinski signs, profound sensory loss below the T9 level, and marked tenderness over the thoracolumbar spine.

Laboratory evaluation demonstrated normocytic normochromic anemia (hemoglobin 9.6 g/dL), leukocytosis (13.94 × 10³/µL) with thrombocytosis, hypoalbuminemia (2.86 g/dL), hypoproteinemia, and mild renal dysfunction (serum creatinine 1.26 mg/dL), which resolved after intravenous fluid administration. Human immunodeficiency virus (HIV), hepatitis B surface antigen (HBsAg), and hepatitis C virus (HCV) antibody testing were non-reactive. Additional laboratory findings are summarized in Table 1.

**Table 1:** Laboratory findings on admission and follow-up. HIV, human immunodeficiency virus; HBsAg, hepatitis B surface antigen; anti-HCV, hepatitis C virus antibodies; AFB, acid-fast bacilli; PSA, prostate-specific antigen; CEA, carcinoembryonic antigen; AFP, alpha-fetoprotein; CA 19-9, carbohydrate antigen 19-9; ESR, erythrocyte sedimentation rate; CRP, C-reactive protein.

Parameter	Admission	Follow-up	Reference range
Hemoglobin (g/dL)	9.6	9.6	12.0 – 18.0
Hematocrit (%)	29.1	30.1	37 – 51
Leukocytes (×10³/µL)	13.94	5.65	5.0 – 10.0
Platelets (×10³/µL)	584	318	150 – 450
Sodium (mmol/L)	130.4	128.1	135 – 148
Glucose (mg/dL)	113	77.9	70 – 105
Urea (mg/dL)	57.7	39.5	10 – 55
Creatinine (mg/dL)	1.26	0.80	0.60 – 1.10
Albumin (g/dL)	2.86	—	3.5 – 5.0
Total protein (g/dL)	5.29	—	6.0 – 8.4
HIV	Non-reactive	—	Non-reactive
HBsAg	Non-reactive	—	Non-reactive
Anti-HCV antibodies	Non-reactive	—	Non-reactive
Sputum AFB smear	Negative	Negative	Negative
Total PSA (ng/mL)	0.11	—	0 – 4.0
CEA (ng/mL)	0.68	—	0 – 4.1
AFP (IU/mL)	0.64	—	0 – 5
ESR (mm/h)	--	98	0 – 20
CRP (mg/L)	--	34	<5

Computed tomography (CT) of the thorax, abdomen, and pelvis revealed multifocal osteolytic lesions involving the axial skeleton, including the sternum, thoracic and lumbar vertebrae, and iliac bones (Figure 1). Given the multifocal skeletal distribution, disseminated metastatic disease was initially considered the leading diagnostic possibility. However, serum tumor markers, including prostate-specific antigen, carcinoembryonic antigen, alpha-fetoprotein, and carbohydrate antigen 19-9, were within normal limits. Serial sputum acid-fast bacilli smears, blood cultures, and urine cultures were negative (Table 1).

**Figure 1:**
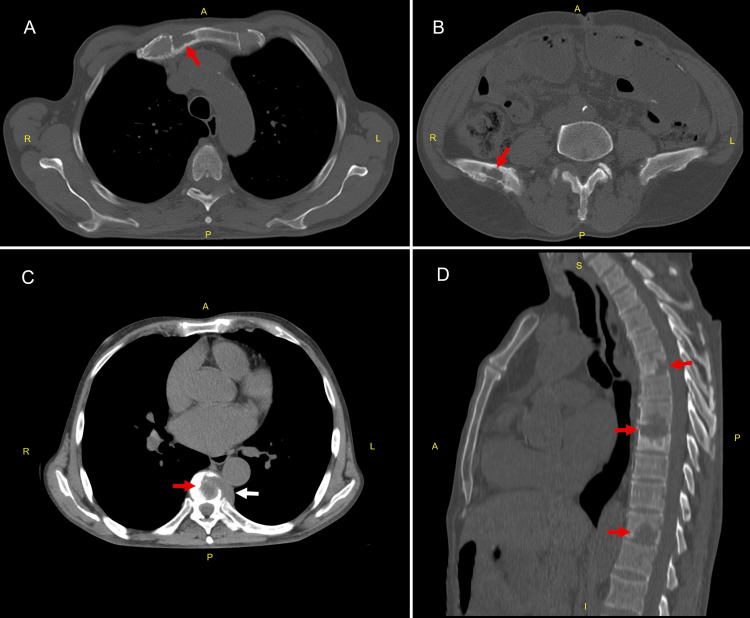
CT findings of multifocal skeletal tuberculosis initially suggestive of disseminated metastatic disease. (A) Axial bone-window CT image demonstrating an osteolytic lesion involving the sternum (red arrow). (B) Axial CT image showing an osteolytic lesion involving the right iliac bone (red arrow). (C) Axial CT image demonstrating a lytic lesion involving a thoracic vertebral body with cortical disruption (red arrow) and an associated left paravertebral soft-tissue extension (white arrow). (D) Sagittal CT reconstruction demonstrating multiple noncontiguous vertebral osteolytic lesions (red arrows). CT, computed tomography. Orientation markers: A, anterior; P, posterior; S, superior; I, inferior; R, right; L, left.

Magnetic resonance imaging (MRI) of the thoracic spine, including short tau inversion recovery (STIR), post-contrast T1-weighted, and T2-weighted sequences, demonstrated destructive tuberculous spondylodiscitis with associated epidural and paravertebral extension resulting in spinal canal compromise (Figure 2).

**Figure 2:**
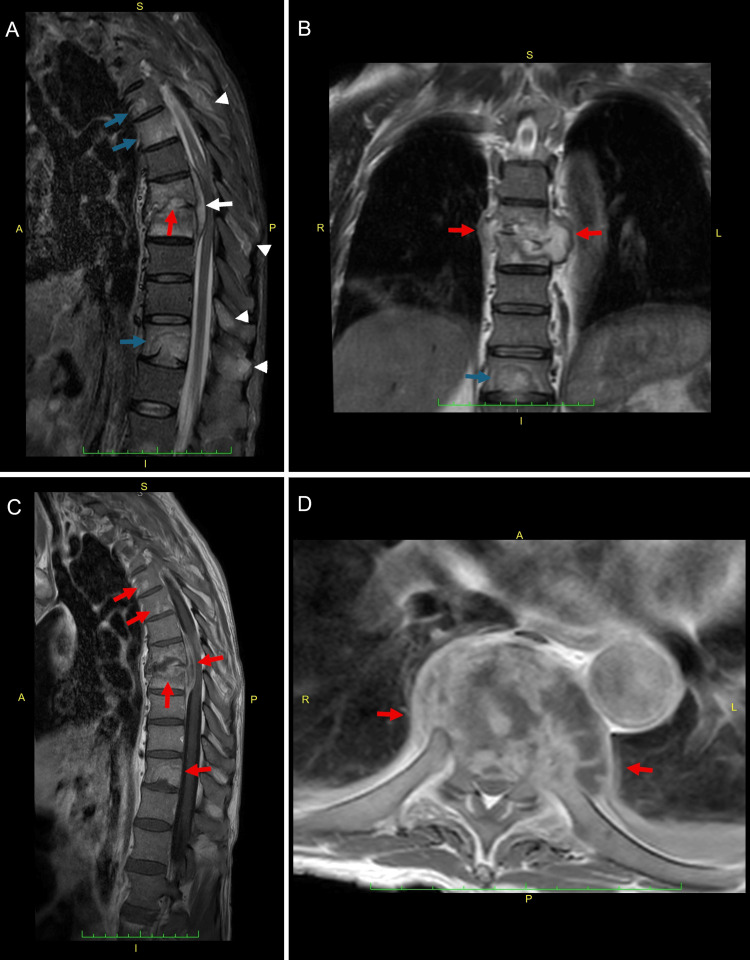
MRI of the thoracic spine. (A) Sagittal STIR image demonstrating marked bone marrow edema and destruction/erosion of the vertebral endplates at the T7-T8 level, with a predominance in the inferior endplate of T7, alongside involvement of the intervertebral disc and marked narrowing of the intersomatic space (red arrow). Posterior epidural soft-tissue extension causing indentation and compression of the thecal sac is also identified (white arrow). Additional noncontiguous vertebral marrow edema involving upper and lower thoracic vertebral bodies is observed (blue arrows). Inflammatory involvement extending posteriorly into the spinous processes is highlighted (white arrowheads). (B) Coronal T2-weighted image demonstrating involvement of the T7-T8 vertebral level with associated paravertebral collections extending bilaterally (red arrows) and a separate noncontiguous focus at T11 (blue arrow). (C) Sagittal post-contrast T1-weighted image demonstrating prominent, multifocal enhancement of both contiguous and noncontiguous vertebral lesions, as well as peripheral rim enhancement of the associated epidural lesion (red arrows).(D) Axial post-contrast T1-weighted image showing paravertebral soft-tissue extension adjacent to the affected thoracic vertebral body with peripheral rim enhancement on both sides (red arrows). MRI, magnetic resonance imaging; STIR, short tau inversion recovery. Orientation markers: A, anterior; P, posterior; S, superior; I, inferior; R, right; L, left. Green scale bars at the bottom of each panel indicate metric calibration for anatomical dimensions.

Given the diagnostic uncertainty, additional laboratory investigations were performed, revealing elevated inflammatory markers (Table 1). A CT-guided percutaneous biopsy of an affected vertebral lesion was subsequently performed. Conventional polymerase chain reaction (PCR) performed on the biopsy specimen detected *Mycobacterium tuberculosis *complex, establishing the definitive diagnosis of multifocal skeletal tuberculosis involving the spine, sternum, and iliac bones.

The patient was evaluated by the Infectious Diseases service, which initiated antituberculous therapy with isoniazid, rifampin, pyrazinamide, and ethambutol. The planned regimen consists of a two-month intensive phase followed by a 10-month continuation phase with isoniazid and rifampin, for a total treatment duration of 12 months. Subsequently, the patient was evaluated by the Neurosurgery service, where he was deemed a candidate for surgical decompression and is currently undergoing preoperative assessment.

## Discussion

Establishing a diagnosis of multifocal skeletal tuberculosis may be challenging because clinical manifestations are often nonspecific and conventional microbiological studies frequently yield negative results. In the present case, acid-fast bacilli smears, blood cultures, urine cultures, and tumor markers were unremarkable despite extensive skeletal involvement. Definitive diagnosis was established through CT-guided biopsy followed by conventional PCR, which identified *Mycobacterium tuberculosis *complex. Recent studies have demonstrated the high diagnostic accuracy of molecular techniques and the utility of image-guided tissue sampling for establishing a microbiological diagnosis when conventional diagnostic methods are inconclusive. Although newer molecular platforms such as Xpert MTB/RIF Ultra (Cepheid, Sunnyvale, USA) have shown excellent diagnostic performance, conventional PCR may still provide rapid and clinically meaningful confirmation when more advanced molecular testing is unavailable [6,7].

The most distinctive feature of this case was the multifocal pattern of skeletal involvement. The combination of constitutional symptoms and multiple osteolytic lesions involving the sternum, vertebral bodies, and iliac bones strongly suggested an underlying malignant process. The presence of both sternal and iliac involvement further increased diagnostic uncertainty, and normal tumor marker levels did not exclude a neoplastic etiology. Similar diagnostic challenges have been reported in previous cases in which radiological findings initially suggested metastatic malignancy and contributed to delayed diagnosis and treatment [8-11].

Although the multifocal distribution represented the most distinctive aspect of this case, vertebral involvement was responsible for the most severe clinical consequences. Progressive vertebral destruction with epidural extension resulted in spinal cord compression, culminating in paraplegia and urinary retention. Neurological deficits remain among the most serious consequences of spinal involvement, particularly in patients with delayed diagnosis and advanced disease at presentation [3]. While antituberculous therapy remains the cornerstone of treatment, surgical intervention may be required in selected patients with neurological compromise, spinal instability, or persistent compression of neural structures [12]. 

This case highlights the broad clinical spectrum of skeletal tuberculosis and its ability to mimic neoplastic disease. Multifocal skeletal tuberculosis may present with osteolytic lesions that are initially interpreted as metastatic malignancy on advanced imaging studies [8-11]. This radiological overlap is particularly challenging because both entities may demonstrate multifocal skeletal involvement, cortical destruction, and soft-tissue extension, making differentiation based on imaging findings alone difficult. Maintaining a high index of suspicion for tuberculosis in patients with multifocal osteolytic lesions, constitutional symptoms, and progressive neurological deficits is essential, particularly in endemic regions. Early tissue diagnosis and molecular confirmation remain critical for avoiding delays in treatment and potentially irreversible neurological sequelae.

A limitation of this case is that histopathological examination and mycobacterial culture results were not available; therefore, diagnosis relied on molecular confirmation by PCR. Nevertheless, molecular confirmation from the biopsy specimen, together with the clinical and radiological findings, supported the diagnosis of multifocal skeletal tuberculosis. In addition, long-term follow-up data after initiation of antituberculous therapy were not available at the time of manuscript preparation, precluding assessment of neurological recovery, radiological response, and long-term functional outcomes.

## Conclusions

Multifocal skeletal tuberculosis may closely mimic metastatic malignancy, particularly in patients presenting with constitutional symptoms and multiple osteolytic lesions. This case highlights the importance of maintaining a high index of suspicion for tuberculosis in the differential diagnosis of multifocal skeletal involvement and underscores the value of tissue sampling combined with molecular testing for establishing a definitive diagnosis. Although conclusions from a single case should be interpreted with caution, this report emphasizes the potential for delayed diagnosis and severe neurological complications when multifocal skeletal tuberculosis is not promptly recognized.
